# Who Cares? Examining Marriage, Culture, and Family Approval on Perceived Obligations to Provide Dementia Care

**DOI:** 10.1093/geroni/igaf122.3327

**Published:** 2025-12-31

**Authors:** Tim Killian, Allison Martin

**Affiliations:** University of Arkansas, Fayetteville, Arkansas, United States; University of Arkasnas, Fayetteville, Arkansas, United States

## Abstract

Dementia care presents significant challenges for family caregivers, whose perceived obligations are shaped by relational, cultural, and psychosocial factors. This study examines variations in caregiving expectations based on marital status, marital history, gender, sexual intimacy, and adult children’s approval. Additionally, we explore how broader cultural values, including familism and individualism, shape perceived caregiving responsibilities. Using a nationally representative Qualtrics sample of 300 U.S. adults aged 55 and older, participants responded to vignettes depicting caregiving scenarios. Data were first analyzed using descriptive statistics and correlation analysis. Next, mixed-effect multiple regression models were used to assess predictors of perceived caregiving obligations while accounting for individual differences. Results indicate that marital status plays a predicted perceived obligations, with married partners having significantly greater caregiving obligations than those in non-marital relationships (p < 0.0001). Familism was a strong predictor of caregiving expectations (p < 0.0001), reinforcing caregiving as a cultural obligation. However, findings did not support the assumption that sexual intimacy significantly influences caregiving expectations (p = 0.30). Additionally, adult children’s approval had only marginal effects on caregiving perceptions (p = 0.08), suggesting that while intergenerational relationships may shape caregiving roles, they are not the primary determinant. These findings have implications for policy and intervention strategies aimed at supporting family caregivers, particularly in addressing the needs of diverse family structures and cultural backgrounds. Future research should further explore further the roles of cultural values and personal beliefs in shaping caregiving responsibilities.

